# A Survey of Double Contrast Barium Enemas at Bristol Royal Infirmary

**Published:** 1983-10

**Authors:** P. G. P. Stoddart, J. Virjee

**Affiliations:** Senior Registrar and Consultant Radiologist, Bristol Royal Infirmary; Senior Registrar and Consultant Radiologist, Bristol Royal Infirmary


### A Survey of Double Contrast Barium Enemas at Bristol Royal Infirmary

**P. G. P. Stoddart, D.M.R.D. and J. Virjee, F.R.C.R.**
Senior Registrar and Consultant Radiologist, Bristol Royal Infirmary

### INTRODUCTION

Barium enemas are one of the most commonly requested radiological examinations. Here in Bristol the enemas are carried out in double contrast, which is becoming widely accepted as the standard examination of the colon (Thoeni and Margulis 1978). However there are several problems which have to be overcome if an optimal double contrast examination is to be obtained.

The colon must be free of its contents if abnormalities are not to be obscured, although this is true of all bowel examinations (Laufer 1976). Good mucosal coating must be obtained if fine details are to be seen. The radiologist's technique must be competent so that all the colon is seen in double contrast, preferably in at least two projections. Finally the examination is of no value unless it is interpreted accurately.

Five hundred barium enemas were reviewed to identify how faecal contamination, poor mucosal coating, inadequate radiological technique, and poor interpretation detracted from the accuracy of the examination.

### PATIENTS AND METHODS

All the patients were referred from the wards or out-patient clinics of the Bristol Royal Infirmary. The enemas were only reviewed if the bowel was in continuity from the caecum to the anus. They were rejected from the series if a repeat examination had been recommended because of heavy faecal contamination or poor radiological technique.

All the patients had the same bowel preparation involving 2 days of a low residue diet, laxatives, and pre-examination colonic lavage. They were examined by a standard technique (Chapman and Nakielny, 1981). The examinations were carried out by 4 consultants, 10 senior registrars and 19 registrars. The reports were checked and contersigned by 4 consultants and 7 senior registrars.

The enemas were reviewed without seeing the original reports. The pathological findings were recorded, and the examinations were assessed for faecal contamination, mucosal coating, and radiological technique according to the criteria listed in Table 1. Finally the review findings were compared with the original report. When these differed the enema was checked at a later date, and it was recorded which report was deemed correct.

**Table 1**

| | |
|---|---|
| Faecal assessment | |
| Good | Clean |
| Adequate | Some residue, none of the colon obscured |
| Poor | Part of the colon obscured |
| Mucosal coating assessment | |
| Good | Well coated throughout the colon |
| Adequate | Suboptimal but considered diagnostic |
| Poor | Extensive mucous smearing, or diagnostically inadequate coating |
| Technical assessment | |
| Good | All of the colon seen in double contrast |
| Adequate | 80% of the colon seen in double contrast |
| Poor | Less than 80% seen in double contrast |

### RESULTS

### A. FAECAL AND MUCOSAL ASSESSMENTS

The assessments of faecal contamination and mucosal coating are summarised in Table 2. The 84 ward patients were more likely to have a poor faecal assessment ($P<0.01$) and poor mucosal coating ($P<0.05$). Large bowel abnormalities were found in more ward patients (71%) than outpatients (47%), this difference having a significance of $P<0.001$.

**Table 2**

| | | Good (%) | Adequate (%) | Poor (%) |
|---|---|---|---|---|
| Faeces | | | | |
| All patients | (500) | 60 | 22 | 18 |
| In patients | (84) | 52 | 19 | 29 |
| Out patients | (416) | 61 | 23 | 16 |
| Cases with poor technique | (44) | 34 | 27 | 39 |
| Mucosa | | | | |
| All patients | (500) | 57 | 32 | 11 |
| In patients | (84) | 46 | 36 | 18 |
| Out patients | (416) | 59 | 31 | 10 |
| Cases with poor technique | (44) | 23 | 50 | 27 |

**Table 3**

| | Good | Adequate | Poor |
|---|---|---|---|
| Assessments in 61 cases of polyps | | | |
| Faecal contamination | 38 | 14 | 9 |
| Mucosal coating | 40 | 18 | 3 |
| Assessments in 31 cases of inflammatory bowel disease | | | |
| Faecal contamination | 21 | 7 | 3 |
| Mucosal coating | 24 | 3 | 4 |

The faecal and mucosal assessments in the 31 cases of inflammatory bowel disease and the 61 cases in which polyps were found are summarised in Table 3. Polyps ($P<0.05$) and inflammatory bowel disease ($P<0.05$) were seen more frequently in patients with good mucosal coating. Both were also seen relatively often in patients with a good faecal assessment.

**B. TECHNIQUE**

The assessments of the radiological technique are summarised in Table 4. There is no correlation between the experience of the radiologist and the proportion of poor examinations. Patients on whom a technically poor examination was performed were more likely to have a poor faecal assessment ($P<0.001$) and poor mucosal coating ($P<0.01$) (see Table 2).

**C. REPORTING**

There were 55 cases, summarised in Table 5, in which the original report and the review findings

**Table 4**

**Radiologists Technique**

| | No. of cases | Good (%) | Adequate (%) | Poor (%) |
|---|---|---|---|---|
| Consultant | 52 | 54 | 35 | 11 |
| Senior Registrar | 146 | 64 | 28 | 8 |
| Third year Registrar | 83 | 52 | 40 | 8 |
| Second year Registrar | 163 | 59 | 32 | 9 |
| First year Registrar | 56 | 64 | 29 | 7 |
| All cases | 500 | 59 | 32 | 9 |

differed. On checking these cases we found that many of the discrepancies were due to observer error. However in some cases the difference was one of interpretation of the signs. In these cases it was decided which interpretation was most probably correct.

In 42 cases the original report was deemed to be inaccurate. Twenty-eight of these cases had been countersigned by a consultant (6.7% of 416 reports), and 14 had been countersigned by a senior registrar (16.7% of 84 reports). The difference between the consultant and the senior registrar rates has a significance of $P<0.01$. In 13 cases the review was deemed to be inaccurate (2.6% of 500 cases).

### DISCUSSION

These enemas were performed in a teaching department, the majority being carried out by junior staff. This might be expected to result in a poor technical standard overall. However there was no correlation between the experience of the radiologist and the technical quality of the enemas. This is a reflection both of the supervision that the junior trainees receive, and of the reservation of the more difficult cases for the senior staff. It also agrees with the findings of Campbell, who found that all barium studies of the bowel could be carried out by radiographers under supervision, without loss of diagnostic accuracy (Campbell et al. 1969).

Another potential problem in a teaching department is the issuing of reports by relatively junior staff. This was found in this series, there being a difference in the number of reporting errors by the senior registrars (16.7%) and the consultants (6.7%). However there were only two serious diagnoses that were not reported. These were a carcinoma in the original reports, and a carinoma in the review. Both of these cases were reported by consultants. While this is two cases too many, it would be unrealistic to

**Table 5**

**Cases of Observer Discrepancy**

| | *Diverticula* | *Polyp* | *Inflammatory disease* | *Carcinoma* | *Extrinsic mass* | *Stricture* | *Total (%)* |
|---|---|---|---|---|---|---|---|
| Original reports | | | | | | | |
| Pathology missed: | | | | | | | |
| Consultant (416 cases) | 9 | 10 | 4 | 1 | 1 | 0 | 6 |
| Senior registrar (84 cases) | 3 | 3 | 3 | 0 | 0 | 0 | 10.7 |
| Pathology wrongly reported | | | | | | | |
| Consultant (416 cases) | 2 | 1 | 0 | 0 | 0 | 0 | 0.7 |
| Senior registrar (84 cases) | 0 | 5 | 0 | 0 | 0 | 0 | 6 |
| Review reports | | | | | | | |
| Pathology missed (500 cases) | 2 | 6 | 1 | 1 | 0 | 0 | 2 |
| Pathology wrongly reported (500 cases) | 0 | 1 | 1 | 0 | 0 | 1 | 0.6 |

expect to never miss anything. Saunders found a similar rate of one colonic tumour missed in every 500 examinations because of radiological mistakes (Saunders and MacEwen, 1971).

The difference in the number of 'errors' in the original reports and in the review is largely artefactual. When checking the discrepant reports it was often a matter of interpretation or opinion as to which was most correct. It is therefore not surprising that the final opinion more often reflected the author's review findings than the original report. The error rate in the review (2.6%) is probably close to the real error rate in the department.

This survey has highlighted ward patients as those most likely to cause diagnostic difficulties. Both their faecal and mucosal assessments were consistently worse than the out-patients'. In addition the patients with poor bowel preparation were more likely to have a technically poor examination. The reasons for this are probably multiple. The compliance of the ward patients to the 48 hour low residue diet is probably better than that of the out-patients. However the ward patients are relatively sedentary, and are in relatively poor clinical condition. Both these factors will tend to lead to the colon becoming loaded with faeces. Furthermore many more ward patients had large bowel disease.

### SUMMARY

This survey has shown that trainee radiologists are able to master satisfactorily the technique of double contrast barium enemas. However the enemas are not interpreted as accurately by the junior staff as by the consultants. Both poor mucosal coating and faecal contamination detracted from the accuracy of the examinations, although poor mucosal coating was the greater problem. Both occurred more frequently in ward patients, and in patients on whom a technically poor examination had been carried out.

### ACKNOWLEDGEMENTS

We would like to thank Mrs. J. Wilkinson (Superintendent Radiographer) and Miss Nicola Eberle for their help and encouragement.

### REFERENCES

CAMPBELL, J. A., LIEBERMAN, M., MILLER, R. E., DREESEN, R. G. and HOOVER, C. (1969). Experience with technician performance of gastrointestinal examinations. *Radiology* **92**, 65–73.

CHAPMAN, S. and NAKIELNY, R. (1981). A guide to radiological procedures. London, Bailliere Tindall, pp. 28–32.

LAUFER, I. (1976) The double contrast barium enema: myths and misconceptions. *Gastrointestinal Radiology* **1**, 19–31.

SAUNDERS, C. G. and MacEWEN, D. W. (1971) Delay in diagnosis of colonic cancer – a continuing challenge. *Radiology* **101**, 207–208.

THOENI, R. F. and MARGULIS, A. R. (1978) The state of radiographic technique in the examination of the colon – a survey. *Radiology* **127**, 317–323.

